# Intergenerational Wisdom Exchange Program (WisE): Reducing social isolation for Black and Hispanic older adults

**DOI:** 10.1093/geroni/igaf122.3612

**Published:** 2025-12-31

**Authors:** Athena Chan, Rodlescia Sneed

**Affiliations:** Texas Tech University, Lubbock, Texas, United States; Wayne State University, Detroit, Michigan, United States

## Abstract

Black and Hispanic older adults face disproportionately high rates of social isolation and loneliness, yet culturally tailored interventions remain scarce. The Intergenerational Wisdom Exchange Program (WisE) addresses this gap through a community-engaged initiative that pairs college students with socially isolated Black and Hispanic older adults in an 8-week program focused on reciprocal learning. College students share digital literacy skills, while older adults share their life stories, creating opportunities for meaningful intergenerational exchange. A Community Advisory Board of Black and Hispanic older adults and college students guides the design, refinement, and cultural tailoring of WisE. This collaborative process informs program structure, builds trust, and addresses barriers to participation, ensuring reminiscence and digital literacy remain meaningful and empowering. In this presentation, we describe how community engagement shapes WisE’s development, highlighting lessons learned from iterative feedback and co-design with community members. We discuss how the advisory board influences recruitment strategies, session activities, and approaches to fostering intergenerational connection. Through this process, we demonstrate that combining reminiscence with digital literacy supports meaningful intergenerational relationships, reduces social isolation, and promotes psychosocial well-being. WisE illustrates how community-guided design strengthens intervention relevance and sustainability. By centering the voices of Black and Hispanic older adults and students, we create a sustainable model that can be adapted and replicated to address social isolation and loneliness in diverse communities.

